# Recall, perceptions and determinants of receiving physical activity advice amongst cancer survivors: a mixed-methods survey

**DOI:** 10.1007/s00520-021-06221-w

**Published:** 2021-04-22

**Authors:** Samuel T. Orange, Stephen E. Gilbert, Morven C. Brown, John M. Saxton

**Affiliations:** 1 School of Biomedical, Nutritional, and Sport Sciences, Faculty of Medical Sciences, Newcastle University Centre for Cancer, Newcastle University, Newcastle upon Tyne, UK; 2 Institute for Musculoskeletal Health, The University of Sydney and Sydney Local Health District, Sydney, Australia; 3 Faculty of Medical Sciences, Population Health Sciences Institute, Newcastle University Centre for Cancer, Newcastle University, Newcastle upon Tyne, UK; 4 Department of Sport, Health & Exercise Science, University of Hull, Cottingham Road, Hull, HU6 7RX UK

**Keywords:** Physical activity advice, Cancer survivors, Mixed-methods survey

## Abstract

**Purpose:**

This study explored cancer survivors’ views and experiences of receiving physical activity advice post-diagnosis. We also determined the influence of sociodemographic characteristics on the recall of physical activity advice and whether receiving advice was associated with meeting physical activity guidelines.

**Methods:**

An anonymised, mixed-methods, 27-item survey was distributed to cancer survivors via online cancer communities in the UK.

**Results:**

Of the 242 respondents, 52% recalled receiving physical activity advice. Of those who recalled receiving advice, only 30% received guidance on type of physical activity and 14% were referred to another source of information or exercise specialist. Advice was most often given after treatment cessation, with only 19% of respondents receiving advice during active treatment. Most respondents (56%) expressed a need for further information. There was no evidence of associations between sociodemographic characteristics and recall of physical activity advice. However, cancer survivors who perceived the physical activity advice they received as being appropriate (odds ratio [OR] 3.8, 95% confidence interval [95% CI]: 1.4–10.7) and those with a higher level of education (OR 3.2, 95% CI: 1.8–5.8) were more likely to meet aerobic exercise guidelines. Females were less likely to meet resistance exercise guidelines than males (OR 0.44, 95% CI: 0.21–0.90).

**Conclusion:**

There is scope to improve the provision of physical activity advice in cancer care by providing advice in a timely manner after diagnosis, referring patients to a suitable exercise or rehabilitation specialist when indicated, and using a tailored approach to ensure the advice is appropriate for specific sociodemographic groups.

**Supplementary Information:**

The online version contains supplementary material available at 10.1007/s00520-021-06221-w.

## Introduction

A growing body of evidence supports the role of physical activity (including planned, structured exercise) as an adjunct therapy in cancer care. Data from randomised controlled trials (RCTs) show that physical activity attenuates some adverse side effects of cancer and cancer treatment [1, 2]. Epidemiological data also show that post-diagnosis physical activity is associated with a reduced risk of cancer recurrence and mortality in colon, breast, and prostate cancers [3, 4]. This evidence has given rise to the development of physical activity guidelines for cancer survivors, with the American College of Sports Medicine (ACSM) recommending that cancer survivors engage in at least 30 min of moderate-intensity aerobic exercise on at least 3 days per week, as well as twice-weekly resistance exercise [2].

Despite the established health benefits of physical activity, the proportion of cancer survivors living a physically active lifestyle in the UK remains low. A national survey of 3300 breast, colorectal, non-Hodgkin lymphoma, and prostate cancer survivors showed that less than half (45%) met current ACSM physical activity guidelines for cancer survivors, and 31% of respondents were completely inactive [5]. The Living with and Beyond Colorectal Cancer survey also reported that one in three colorectal cancer survivors did not perform any physical activity [6]. Clearly, strategies are required to translate physical activity recommendations into accessible and sustainable interventions that can have a long-lasting impact on the quality of cancer survivorship.

The ACSM recently proposed the ‘Moving Through Cancer’ initiative to increase the proportion of cancer survivors who are physically active [7, 8]. This involves healthcare professionals assessing, advising, and referring patients to exercise services. Receiving an exercise recommendation from an oncologist has been shown to increase exercise behaviour in newly diagnosed breast cancer survivors, compared with a control group receiving no recommendation [9]. However, discussing physical activity with cancer survivors is not yet standard practise within oncology care [10, 11]. Furthermore, there is evidence that only one in three cancer survivors recalls receiving physical activity advice from a healthcare professional [6, 12]. Gaining a better understanding of how survivors are likely to perceive and respond to physical activity advice could improve patient-physician discussions of physical activity, as well as informing the design of patient-centred interventions to promote regular physical activity.

Cancer survivors’ views and experiences of receiving physical activity advice are not well understood. A small number of studies have collected qualitative information on the support needs of survivors in relation to health-promoting behaviours [13, 14]. However, to our knowledge, no previous research has explored cancer survivors’ perceptions of physical activity advice in terms of the type of advice received, when and from whom they received it, and its perceived appropriateness and sufficiency. There is also a lack of information regarding how patient sociodemographic factors influence physical activity behaviour and the recall of physical activity advice amongst cancer survivors. This information is important to inform and guide the provision of physical activity advice in oncology settings so that healthcare professionals can engage specific sociodemographic groups and help tackle health inequalities that typically exist in the general population [15].

As a means of addressing the call for more research into contextual issues surrounding the provision of physical activity advice within the cancer care pathway [6], this study aimed to explore cancer survivors’ views and experiences of receiving physical activity advice after their cancer diagnosis. We also aimed to determine the influence of sociodemographic characteristics on the recall of physical activity advice and whether receiving advice was associated with meeting physical activity guidelines.

## Methods

### Study design

This study used a cross-sectional, mixed-methods survey design. Participants anonymously completed the online-based survey after reading an information document and providing their informed consent. The study was approved by the Faculty of Health and Life Sciences Ethics Committee at Northumbria University.

### Participants and recruitment

A link to the online questionnaire, along with an introduction to the study, was distributed via various UK-based online cancer communities from February to October 2017. Cancer survivors frequently engage with online cancer communities as platforms for information exchange and source of support [16–18]. Permission was obtained from the website owners before posting the link online. Eligible participants were cancer survivors who were aged ≥ 18 years and able to understand written instructions in English. Participants reporting a previous diagnosis of dementia were excluded.

### Measures

The survey was created using a web-based survey application (Google Forms, Google LLC, CA, USA) and comprised of 27 items relating to: (i) sociodemographics, (ii) cancer diagnosis and treatment, (iii) current physical activity behaviour, and (iv) views and experiences of physical activity advice. Index of multiple deprivation scores were calculated for the home postcode and are presented as deciles (1–10 from least to most deprived). Feedback was sought from the North East and North Cumbria Public Involvement Consumer Panel before distributing the survey, and survey questions are available on the Open Science Framework (OSF) repository [19].

The self-reported frequency and duration of aerobic and resistance exercise were evaluated using a modified version of the validated Godin Leisure Time Exercise Questionnaire [20]. Aerobic exercise was divided into three intensity domains: low-, moderate-, and high-intensity. The frequency of vigorous-intensity aerobic exercise was assessed by asking: ‘Considering a typical week (7 days) over the past month, how many days on average did you do vigorous intensity aerobic exercise (heart beats rapidly, sweating) e.g. running, aerobics classes, vigorous swimming, vigorous cycling?’ This was followed up with a question about the duration of activity: ‘What would be the average duration of any vigorous intensity aerobic sessions you take part in?’ These questions were then repeated for moderate- and low-intensity aerobic exercise and for resistance exercise. Participants were considered to be adhering to aerobic exercise guidelines for cancer survivors if they reported performing at least 30 min of moderate- to vigorous-intensity aerobic exercise on at least 3 days per week [2]. Adherence to resistance exercise guidelines was defined as performing strength/resistance exercise at least two times per week [2]. The perceived change in physical activity participation was also evaluated by asking the close-ended question: ‘How have your physical activity habits changed since your cancer diagnosis?’ Possible responses to this question included ‘I became more active’, ‘I became less active’, ‘My activity habits have no changed’, or ‘I do not know’.

Recall of physical activity advice was assessed with the close-ended question: ‘Has anyone given you advice about physical activity since your cancer diagnosis?’ If the respondent answered ‘Yes’, further close-ended questions were used to gather information on who provided the advice, at what stage of their cancer treatment they received the advice, whether they felt it was appropriate for their stage of treatment and general health, and whether receiving the advice influenced their physical activity habits. The perceived sufficiency of physical activity advice was evaluated by asking: ‘Do you feel you have received sufficient advice about physical activity since your cancer diagnosis?’ Finally, views of physical activity advice were further explored with three open-ended questions:

- If you have received advice, what advice were you given?
- If you would have liked further advice, what would you have liked more advice about?
- Is there anything else you would like to tell us about your current physical activity habits or the advice you have received on physical activity that has not previously been covered in this survey?

### Sample size

Peduzzi and colleagues [21] suggest the following formula to calculate the sample size required for logistic regression models: *n* = 10* k*/*p*, where *k* is the number of predictors in the model, and *p* is the proportion of positive cases. Previous research has shown that 31% of cancer survivors recall receiving physical activity advice [6]. Given six predictor variables in the model (sociodemographic characteristics), the minimum sample size required to evaluate the influence of patient characteristics on the recall of physical activity advice is 194.

### Data analysis

Survey responses to close-ended questions were summarised using descriptive statistics. Continuous data were reported as median and interquartile range (IQR), and categorical data were reported as frequency and proportion. Responses to open-ended questions were analysed using content analysis [22, 23], which began by assigning codes to phrases within the participants’ responses. These codes were inductively derived and captured thoughts or concepts within the data [24]. A coding frame was developed to enable systematic and reliable coding of the data [22]. Two authors independently coded and any discrepancies were resolved through discussion. Similar or related codes were subsequently grouped to create categories; these categories were further collapsed into main and sub-categories [24]. Data were initially coded in Microsoft Excel (Microsoft Corporation, Redmond, WA), and the frequency at which categories occurred were calculated in R (R Foundation for Statistical Computing, Vienna, Austria).

To examine associations between demographic characteristics and recall of receiving physical activity advice, variables were dichotomised prior to analysis and coded as a binary number (i.e. ‘0′ or ‘1′), although full descriptive data are presented in the results. Recall of physical activity advice was dichotomised as ‘advice’ vs. ‘no advice/not sure’. Demographic characteristics and the binary code for each variable included ethnicity (‘White British’ vs. ‘other’), age (‘ < 60 years’ vs. ‘ ≥ 60 years’), IMD decile (‘ ≤ 5′ vs. ‘ > 5′ or ‘unknown’), sex (‘Male’ vs. ‘Female’), comorbidity (‘ ≥ 1′ vs. ‘none’), and education status (‘academic degree’ or ‘professional qualification’ vs. ‘other qualifications’). We applied a single generalised linear model specifying a binomial distribution and logit link function [25]. Odds ratios (ORs) and 95% confidence intervals (CIs) were calculated as the exponential of the logit coefficient. All demographic characteristics were included in the model to adjust for the effect of potential confounders.

Similar analyses were conducted to evaluate factors associated with achieving physical activity guidelines, which was coded as ‘achieving guidelines’ vs. ‘not achieving guidelines’. Independent variables included the demographic characteristics listed above as well as recall of physical activity advice and perceiving physical activity advice to be appropriate (‘appropriate’ vs. ‘not appropriate/not sure/did not receive advice’). Separate models were applied for aerobic and resistance exercise guidelines as the dependent variables. The absence of multicollinearity in all models was confirmed by variance inflation factor values of < 4 for independent variables. Data were analysed in R and statistical significance was set at *p* < 0.05. Data and code are available on the OSF repository [19].

## Results

Two hundred and forty-three survey responses were collected. After cleaning the data, we identified one duplicate response, which was removed from the data set prior to analysis. Hence, our analysis is based on responses from 242 cancer survivors (Table 1).

**Table 1:** Respondent characteristics (*n* = 242)

Characteristic	Median [IQR] or number (%)
Sociodemographics	
Age (years)	
< 50	66 (27)
50–59	69 (39)
60–69	67 (28)
≥ 70	40 (17)
Sex	
Female	168 (69)
Male	74 (31)
Ethnicity	
White British/Irish	222 (92)
Other White background	10 (4)
Other/prefer not to say	10 (4)
IMD decile	
1–2 most deprived	15 (6)
3–4	24 (10)
5–6	45 (19)
7–8	46 (19)
9–10 least deprived	70 (29)
Unknown	42 (17)
Highest level of education	
O-level/GCSE	26 (11)
High school certificate/A level	18 (7)
NVQ level 1–5 or equivalent	32 (13)
Academic degree	87 (36)
Professional qualification	54 (22)
Other	17 (7)
None	8 (3)
Comorbidities^a^	
Hypertension	45 (19)
Arthritis	23 (10)
CVD	19 (8)
Respiratory disease	18 (7)
Diabetes mellitus (type I or II)	12 (5)
Low back pain	14 (6)
Other	94 (39)
None	111 (46)
Cancer diagnosis and treatment	
Time since diagnosis	
< 6 months	25 (10)
6–12 months	29 (12)
1–2 years	43 (18)
2–5 years	81 (33)
> 5 years	63 (26)
Unknown	1 (0)
Primary cancer	
Breast	92 (38)
Prostate	49 (20)
Colorectal	25 (10)
Ovarian	23 (10)
Oesophageal	15 (6)
Other	38 (16)
Cancer treatment received^a^	
Surgery	184 (76)
Chemotherapy	149 (62)
Radiotherapy	112 (46)
Hormone therapy	79 (33)
Immunotherapy	16 (7)
Not begun	5 (2)
Currently receiving treatment	
Yes	125 (52)
No	117 (48)

*CVD*, cardiovascular disease; *IMD*, index of multiple deprivation; *NVQ*, National Vocational Qualification

^a^Multiple responses are possible

### Recall of physical activity advice

**Fig. 1:**
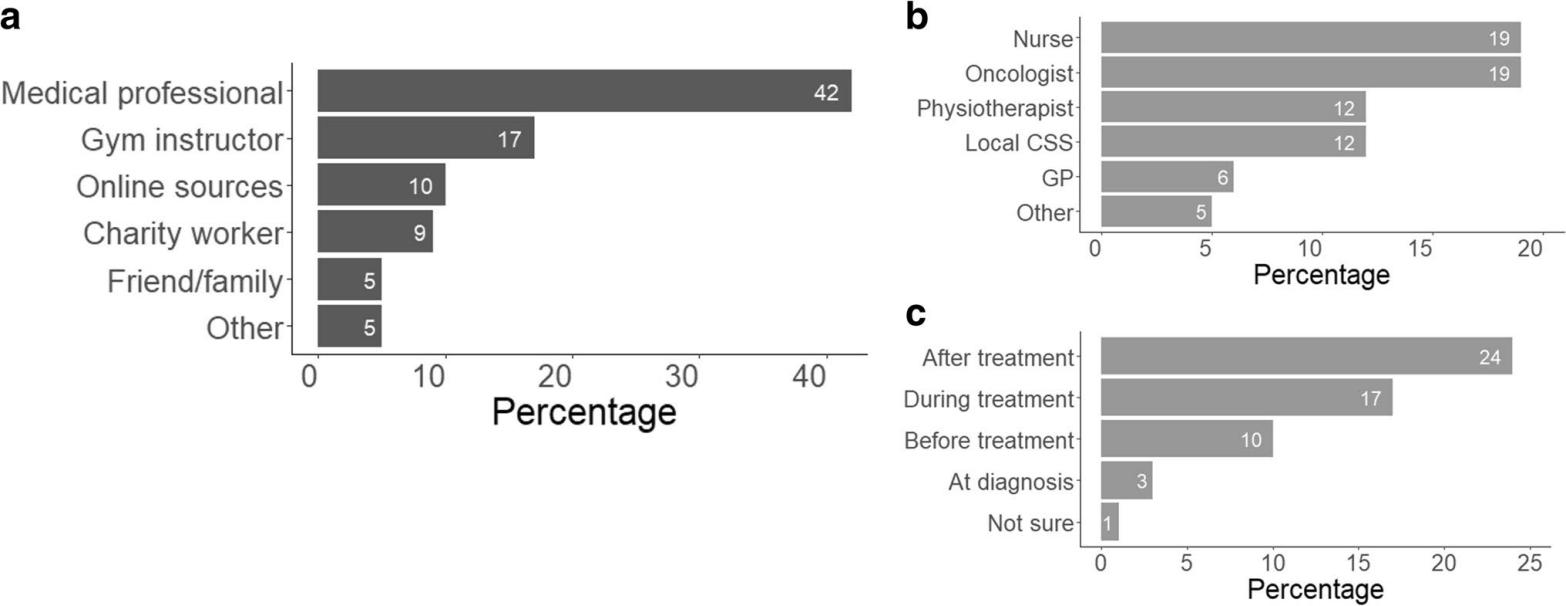
**a** shows who provided the physical activity advice. For participants who recalled receiving advice from a medical professional, **b** shows the type of medical professional who provided the advice, and **c** shows the stage of treatment the advice was given. Multiple responses are possible. Data are presented as a percentage of the 242 respondents. CSS, charity support service; GP, general practitioner

Of the 242 respondents, 52% (*n* = 125) recalled receiving physical activity advice, whilst 48% (*n* = 117) reported not receiving any advice or were not sure. Advice was most commonly received from a medical professional (42%, *n* = 102). When advice was received from a medical professional, it was most often received after cessation of active treatment (24%, *n* = 59) from an oncologist (19%, *n* = 46) and/or nurse (19%, *n* = 47) (Fig. 1). Details of the type of physical activity advice provided to participants are presented in Table 2.

**Table 2:** Physical activity advice received in cancer survivors who recalled receiving advice (*n* = 125)

Type of advice received^a^	Number (%)
Advised to be physically active (general recommendation)	63 (50)
Advised to undertake a specific type of physical activity	38 (30)
Walking/weight bearing exercise	29 (23)
Aerobic exercise	11 (9)
Strength exercise	6 (5)
Yoga/Pilates	5 (4)
Advised to undertake specific characteristics of physical activity	34 (27)
Frequency	23 (18)
Intensity	24 (19)
Duration	16 (13)
Advised to undertake rehabilitation exercises/on how to adapt exercises based on side effects of treatment	16 (13)
Rest or to avoid certain activities	5 (4)
Given access to physical activity resources (e.g. leaflets, group classes) or advised to seek advice from exercise specialist	18 (14)
Advised on the health benefits of physical activity	20 (16)
Other	8 (6)

^a^Multiple responses are possible

### Views of physical activity advice

Of the 125 participants who recalled receiving advice, 82% (*n* = 103) felt it was appropriate based on their stage of treatment and health. Most participants (65%, *n* = 81) reported that receiving advice influenced their physical activity behaviour. Specifically, after receiving physical activity advice, 45% (*n* = 56) said they increased the amount of physical activity they engaged in with or without changing the type of activity, 7% (*n* = 9) said they decreased the amount with or without changing type, and 13% (*n* = 16) reported to change the type of activity only.

Overall, 56% (*n* = 136) of participants said they would have liked more advice about physical activity, 41% (*n* = 99) said the advice they received was sufficient, 1% (*n* = 2) would have liked less advice, and 2% (*n* = 5) were unsure. In particular, patients expressed a need for advice on the type of activity they could undertake (17%, *n* = 42) (see Online Resource 1).

### Additional thoughts

Respondents were given the opportunity to provide additional thoughts on physical activity that was not covered by the survey. Participants said their ability to be physically active after their cancer diagnosis was restricted by treatment-related side effects (14%, *n* = 33), including fatigue (4%, *n* = 10), musculoskeletal pain (5%, *n* = 11) and psychological distress such as low self-esteem (2%, *n* = 5). Five percent (*n* = 11) of respondents wanted referral to an exercise class/programme and 7% (*n* = 17) said that they sought their own advice from resources such as the internet. Seven percent (*n* = 17) emphasised that being physically active during survivorship had benefitted their health.

### Self-reported physical activity

**Fig. 2:**
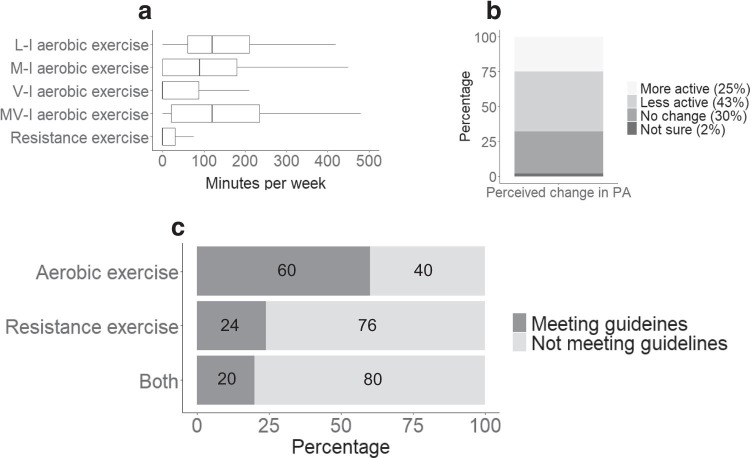
Self-reported levels of physical activity (PA) (**a**), perceived change in PA after cancer diagnosis (**b**), and proportion of respondents meeting physical activity guidelines (**c**). Data are presented as a percentage of the 242 respondents. L-I, low-intensity; M-I, moderate-intensity; V-I, vigorous-intensity; MV-I, combined moderate to vigorous-intensity

The median amount of moderate- to vigorous-intensity aerobic exercise was 145 min·week^−1^ and the median amount of resistance exercise was 25 min·week^−1^ (Fig. 2). Only 20% of participants (*n* = 48) were meeting both aerobic and resistance exercise guidelines. Forty-three percent (*n* = 104) of participants reported that they had become less physically active since their cancer diagnosis.

### Factors associated with recall of advice and meeting physical activity guidelines

**Fig. 3:**
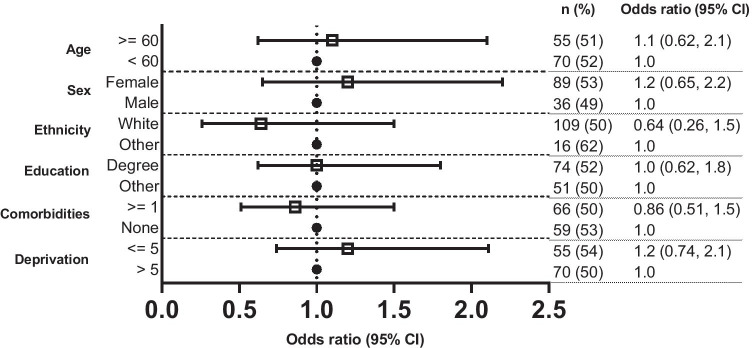
Odds of recalling physical activity advice based on sociodemographic characteristics. The reference category for the dependent variable in the model is ‘not received physical activity advice’. Data are presented as odds ratios with 95% confidence intervals (CIs)

**Fig. 4:**
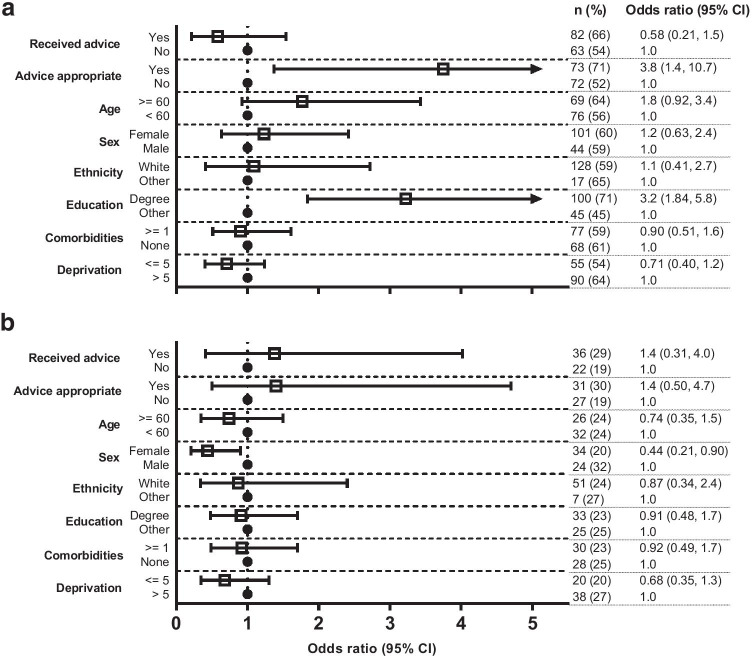
Odds of meeting physical activity guidelines for aerobic (**a**) and resistance (**b**) exercise based on sociodemographic characteristics and recalling physical activity advice. The reference category for the dependent variable in both models is ‘not meeting physical activity guidelines’. Data are presented as odds ratios with 95% confidence intervals (CIs)

There was no evidence of associations between sociodemographic characteristics and the recall of physical activity advice (Fig. 3). However, respondents with a higher level of education (71% vs. 45%; OR 3.2, 95% CI: 1.8–5.8) and those who perceived the physical activity advice they received as being appropriate (71% vs. 52%; OR 3.8, 95% CI: 1.4–10.7) were more likely to meet aerobic exercise guidelines. Respondents who identified as female (vs. male) were less likely to meet resistance exercise guidelines (20% vs. 32%; OR 0.44, 95% CI: 0.21–0.90) (Fig. 4).

## Discussion

This exploratory study provides insight to cancer survivors’ views and experiences of receiving physical activity advice since their cancer diagnosis. Half of the cancer survivors in this study recalled receiving physical activity advice, but only one in five received advice during treatment. Of those who recalled receiving advice, only 30% received guidance on type of physical activity and just 14% were referred to another source of information or exercise specialist. Most respondents (56%) expressed the need for further information. Furthermore, we found that respondents with a higher level of education, and those who perceived the physical activity advice they received as being appropriate, were more likely to meet aerobic exercise guidelines.

The proportion of participants (52%) that recalled receiving physical activity advice is consistent with data from previous studies, reporting proportions in the range of 31–65% [6, 10–12]. A recent survey of 971 oncology clinicians showed that 79% of respondents felt that the treating oncologist should be the person responsible for recommending physical activity to their patients [26]. Thus, our findings provide further evidence that, despite the importance of promoting physical activity being recognised by the oncologist workforce, patient-oncologist discussions of physical activity are not yet standard practise within cancer care.

Our findings extend those of previous studies [6, 10–12] by further exploring contextual factors, including the extent to which advice on specific types of physical activity was provided, and considering the important issues of timing and perceived appropriateness/sufficiency of the advice. Half of the advice given comprised of a general recommendation to be physically active (50%), with less guidance given on type of physical activity to be undertaken (30%). Walking or weight bearing exercise was the most recommended type of activity (23%). Most respondents who received advice perceived it to be appropriate (81%) and said it influenced their physical activity behaviour (62%). Nevertheless, over half of participants expressed a need for more advice (56%), particularly regarding the type and specific characteristics of physical activity (i.e. frequency, intensity, and duration) they could undertake and the associated health benefits, indicating that participants had unmet support needs. This aligns with previous qualitative research demonstrating that oncologists do not adequately address the support and information needs of colorectal and endometrial cancer survivors in relation to physical activity [13, 14].

Providing patients with specific exercise prescriptions is outside the scope of practice for oncology clinicians [7]. Consequently, the ACSM propose that oncologists should promote the importance of physical activity to patients, triage and refer them to an appropriate exercise programme [7, 8]. However, in the present study, only 14% of participants who recalled receiving advice were referred to another source of information or exercise specialist. This is lower than the proportion of oncologists who reported referring patients to an exercise specialist (23%) or providing written information (20%) in a recent survey [27]. Some respondents in this study expressed a need for more information on exercise resources (such as group classes and exercises specialists). The need for educational programmes to ensure that all members of the cancer care team are cognizant of the value of exercise and aware of suitable programmes that patients can be referred to has been highlighted recently [7].

The timing of physical activity advice could also be important to help patients control treatment side effects. Our results show that advice was mostly provided by medical professionals after the cessation of active treatment, whereas only one in five respondents (19%) received advice during treatment. Exercise is generally considered to be safe for cancer survivors during treatment and the requirement for medical clearance in those at low risk of cardiovascular events has also been removed on the basis that it is an unnecessary barrier to participation [2]. However, medical complications associated with locoregional and systemic cancer therapies, as well as other comorbidities, may contraindicate unsupervised exercise. In this case, oncologists may refer patients to outpatient rehabilitation for further evaluation by an appropriately qualified exercise specialist, such as a clinical exercise physiologist or physical therapist. Indeed, 14% of participants in this study said their ability to be physically active was restricted by treatment-related side effects such as fatigue, musculoskeletal pain and low self-esteem, which may require an exercise specialist to prescribe a suitable exercise programme that is adapted to these side effects. This was likely an underestimation because respondents were not specifically asked this question (they raised the issue in response to an open-ended question), and previous research has identified treatment-related side effects and fatigue as key barriers to initiating or maintaining physical activity in cancer survivors [28]. Guidance on how to identify and manage a broad range of cancer-specific exercise contraindications have been published [2, 29, 30].

We did not find evidence of an association between recall of physical activity advice and adherence to physical activity guidelines. Whilst this finding contrasts with previous research reporting a link between receiving physical activity advice and higher physical activity in colorectal cancer patients [6], it is well-established that providing information alone is not sufficient to change health-related behaviour [31]. We did, however, find that cancer survivors with a higher level of education and those who perceived the physical activity advice as being appropriate were more likely to meet aerobic exercise guidelines. This finding aligns with behaviour change interventional research [32] and suggests that simply providing physical activity advice is not enough to influence physical activity behaviour. Research shows that cancer survivors have an interest in being physically active, but preferences and accessibility to participation opportunities vary widely [33]. As such, it is important that healthcare professionals provide physical activity advice that is appropriate for specific sociodemographic groups and is perceived as being acceptable by the patient, which requires an individualised, patient-centred approach. This will likely require the development and dissemination of continuing education training for healthcare professionals regarding tailored delivery of physical activity advice, which is one of the major goals of ACSM’s Moving Through Cancer initiative [8].

Sixty percent of cancer survivors in this study were meeting aerobic exercise guidelines for cancer survivors, whereas just 24% were meeting resistance exercise guidelines [2]. This is reflected in the type of advice given; only 5% of respondents recalled receiving a recommendation to undertake strength-promoting resistance exercise, as opposed to 32% who received advice to walk and/or undergo aerobic exercise. Interestingly, females were less likely to meet resistance exercise guidelines compared with males. This is an important finding given that 9% of advanced solid tumour patients and a quarter cancer patients with obesity are sarcopenic, and convincing evidence that low skeletal muscle mass and sarcopenia adversely impacts cancer survival outcomes [34]. Additionally, because there is strong evidence that resistance exercise alone improves health-related outcomes in cancer survivors [2], further efforts are required to promote adherence to resistance exercise guidelines. Such efforts may have to be adapted to reduce inequality in participation rates between males and females.

This study has some limitations. All information collected in the survey was dependent on patient recall, which is prone to response bias. Self-reported methods of assessing physical activity may also have low validity for assessing incidental or lifestyle physical activity [35]. Twenty-six percent of respondents received their primary cancer diagnosis ≥ 5 years prior, which means that our findings may not be representative of current cancer care. In addition, our findings do not differentiate patients with localised and advanced disease (i.e. different tumour staging, patients suffering from metastatic bone disease, etc.), which could have had some bearing on the results. The possibility of reverse causation is another potential study limitation and as most respondents were Caucasian, our findings may not be generalisable to other ethnic groups. Finally, some of the ORs in this study showed a low level of precision (evidenced by the wide 95% CIs), warranting further research to improve the certainty of estimates and confirm our exploratory findings.

In conclusion, this study showed that physical activity advice is an unmet need for cancer survivors. Most patients in this study expressed their need for further information, particularly regarding the type of physical activity they could undertake and the associated health benefits. Our findings suggest there is scope to improve the provision of physical activity advice in cancer care settings by initiating such discussions in a timely manner after diagnosis, referring patients to a suitable exercise or rehabilitation specialist when indicated, and ensuring the advice is appropriate for specific sociodemographic groups and is considered acceptable by the patient. This is challenging to implement within demanding and financially restricted healthcare systems, but will likely require the dissemination of continuing education training for healthcare professionals with input from multiple stakeholders, including those who will deliver, use, and benefit from the physical activity advice.

## Supplementary Information

Below is the link to the electronic supplementary material.

Supplementary file1 (PDF 30 KB)

## Data Availability

The survey questions and data generated and/or analysed during the current study are available on the Open Science Framework repository (https://osf.io/rj7gz/).

## Code availability

The statistical code from this study is available in the Open Science Framework repository (https://osf.io/rj7gz/).
