# Supplementary material for: Recall, perceptions and determinants of receiving physical activity advice amongst cancer survivors: a mixed-methods survey
Source: Support Care Cancer. 2021 Apr 22;29(11):6369–78. doi: 10.1007/s00520-021-06221-w (PMC8464579; doi:10.1007/s00520-021-06221-w)
Supplement: Supplementary file 1 — Supplementary file1 (PDF 30 KB) [file 520_2021_6221_MOESM1_ESM.pdf]

Online Resource 1. Further advice about physical activity that cancer survivors would have liked to have received (n = 242)

Type of advice[a]	Number (%)
General guidance, reassurance and support	25 (10)
Advise on the type of physical activity	42 (17)
Advise on the frequency, duration, or intensity of physical activity	18 (7)
Advise on when to initiate physical activity	6 (2)
Advise on the benefits of physical activity	24 (10)
Advise on the safety of physical activity	12 (5)
Advise on the Availability of physical activity resources (e.g. leaflets, group classes, exercise specialists)	12 (5)
Advise about what the physical side effects of cancer treatment would be and how to adapt physical activity in light of these side effects	22 (9)
Other	8 (3)

[a]Multiple responses are possible
